# A systematic review on the role of melatonin and its mechanisms on diabetes-related reproductive impairment in non-clinical studies

**DOI:** 10.3389/fendo.2022.1022989

**Published:** 2022-10-11

**Authors:** Maryam Armandeh, Behnaz Bameri, Hamed Haghi-Aminjan, Roham Foroumadi, Mahshid Ataei, Shokoufeh Hassani, Mahedeh Samadi, Mohammad Reza Hooshangi Shayesteh, Mohammad Abdollahi

**Affiliations:** ^1^ Department of Toxicology and Pharmacology, Faculty of Pharmacy, and Toxicology & Diseases Group, Pharmaceutical Sciences Research Center (PSRC), The Institute of Pharmaceutical Sciences (TIPS), Tehran University of Medical Sciences, Tehran, Iran; ^2^ Pharmaceutical Sciences Research Center, Ardabil University of Medical Sciences, Ardabil, Iran; ^3^ Department of Pharmacology, School of Medicine, Tehran University of Medical Sciences, Tehran, Iran; ^4^ Neuroscience Research Center, Iran University of Medical Science, Tehran, Iran; ^5^ Neuroscience Research Center of Qom University of Medical Science, Qom, Iran

**Keywords:** diabetes mellitus, hyperglycemia, melatonin, oxidative stress, genitalia

## Abstract

**Background:**

Diabetes-induced reproductive complications can lead to subfertility and infertility, raising the need to protect reproductive organs. There are limited medications used to improve reproductive health in diabetic patients. Melatonin, mainly produced by the pineal gland, may improve diabetes-associated reproductive complications through various mechanisms and may be a preferred candidate to protect the reproductive system. The present review aims to elucidate the underlying mechanisms of melatonin’s effect on the reproductive system adversely affected by diabetes mellitus (DM).

**Methods:**

A comprehensive systematic literature electronic search was done using the PRISMA guidelines. Web of Science, PubMed, Embase, and Scopus were searched for publications up to June 2022. Search terms were selected based on the study purpose and were explored in titles and abstracts. After screening, out of a total of 169 articles, 14 pertinent articles were included based on our inclusion and exclusion criteria.

**Results:**

The results of studies using rats and mice suggest that DM adversely affects reproductive tissues, including testes and epididymis, prostate, corpus cavernosum, and ovary leading to alterations in histological and biochemical parameters compared to the normal groups. Treatment with melatonin improves oxidative stress, blocks apoptosis induced by endoplasmic reticulum stress and caspase activation, reduces pro-inflammation cytokines, and enhances steroidogenesis.

**Conclusion:**

Melatonin exerted a protective action on the impaired reproductive system in *in-vivo* and *in-vitro* models of DM. The topic has to be followed up in human pregnancy cases that will need more time to be collected and approved.

## 1 Introduction

Diabetes mellitus (DM), a severe metabolic disorder, results from impaired insulin production or dysfunction (1). The vast majority of people with type I diabetes are diagnosed before they reach the age of 30. According to a recent report, type I diabetes is increasing by 3% each year in European children, and an alarming number of children and young adolescents are being diagnosed with type II diabetes (2). DM is one of the most significant stresses in current public health due to its consequences caused by persistent hyperglycemia, including cardiovascular disease, nephropathy, retinopathy, neuropathy, and male and female reproductive injury (3–5). New research concerning the fertility rate in modern communities shows that an increase in the frequency of DM is linked to a decrease in birth and fertility rates. Similar results were also obtained in animal models (6). Recent studies have discovered that DM can be associated with various male reproductive complications, including decreased libido and impotence, erectile dysfunction, abnormal sperm motility and morphology, degenerative and apoptotic alterations in testis, and changes in the levels of hormones, including luteal hormone (LH), follicular stimulating hormone (FSH), and testosterone leading to subfertility and infertility (7). Although the prevalence of reproductive dysfunction in women is lower than in men, some studies have shown a decreased birth rate in diabetic women and reproductive malfunction due to ovarian dysfunction (8).

Moreover, in diabetic women, abnormalities in the menstrual cycle, delayed puberty, amenorrhea, and subsequent infertility have been documented (8–10). The exact mechanisms involved in reproductive dysfunction do not entirely elucidate. The biochemical mechanism underlying the changes in male reproductive capacity caused by DM has been the subject of several clinical and animal research. Endocrine problems, neuropathy, elevated oxidative stress and increased level of advanced glycation end products (AGE) are among them (11, 12). As the studies show, diabetes causes considerable oxidative stress in females, which leads to DNA damage. DNA damage causes cell cycle arrest and cell death, preventing the development of oocytes (13). Hyperglycemia is regarded as one of the major causes of DM-induced complications by activating a range of damaging pathways that appear to be initiated by mitochondrial superoxide overproduction. Transient episodes of hyperglycemia produce tissue damage through processes requiring recurrent acute alterations in cellular metabolism (14). It is well acknowledged that proper diabetes management, namely glycemic control, is critical in minimizing, preventing, or mitigating diabetic complications (15). Nevertheless, routine approved treatments do not reduce long-term issues, and few specific reproductive medications are available for diabetic patients (16, 17). According to recent research, antioxidant therapy lowers the glycemic index, minimizes diabetes complications, and protects against oxidative stress caused by free radicals. It may be a suitable therapeutic option for DM-induced complications (17, 18).

Melatonin (N-acetyl-5-methoxytryptamine) is an indoleamine neurohormone mainly secreted by the pineal gland and locally by several tissues (19, 20). This neurohormone with hydrophilic and lipophilic structure can easily cross all biological barriers and reach a high concentration in intracellular components, including mitochondria, the leading centers for reactive oxygen species (ROS) generation (21, 22). Melatonin exerts several physiological functions such as maintaining the body’s homeostasis, modulation of circadian rhythm, control of neuroendocrine axis, broad-spectrum antioxidant action, reproductive function regulation by affecting steroidogenesis and testicular development, rule of endoplasmic reticulum stress, anti-inflammatory, anti-tumor, anti-aging effect, etc. (23–28).

Oxidative stress induced by many diseases like DM has a prominent role in triggering many complications that can probably be alleviated by the antioxidative effect of melatonin (29). So, this molecule can be a potential candidate for preventing and mitigating DM complications (27).

This review focuses on the role of melatonin in diabetes-induced reproductive injury. Some points include diabetes’ impact on the reproductive system, melatonin’s potential effect on diabetic reproductive dysfunction, and the main mechanisms through which melatonin exerts the possible protective effect or prevents diabetes-induced reproductive dysfunction. So, we will discuss the resulting findings from non-clinical studies using melatonin on the reproductive components in diabetes.

## 2 Methodology

### 2.1 Study protocol

The present systematic review was based on the previous guidelines (30).

### 2.2 Search strategy

A comprehensive literature search was performed in the following electronic databases, including Web of Sciences, PubMed, Scopus, and Embase, with the search terms up to June 2022 without restriction on publication year. The keywords used in this manuscript are in the supplementary file. After eliminating the duplicated studies, firstly, we screened all the studies in the title and abstract according to the abovementioned terms. The study’s full text was included in the second step while meeting our inclusion and exclusion criteria. In the present study, inclusion criteria included 1) the studies based on our aims, 2) the studies with adequate data, 3) there was no limitation in *in-vitro* and *in-vivo* studies, and 4) the studies in the English language. Furthermore, we excluded the studies, which included the following exclusion criteria; 1) case reports, 2) review articles, 3) oral communications, 4) not available articles, 5) letters to the editor, and 6) book chapters. Three reviewers independently performed all the search and screen processes (MA, BB, and HHA). The reviewers excluded the studies that did not meet the present study’s aims.

## 3 Results

### 3.1 Literature search and screening

**Figure 1:**
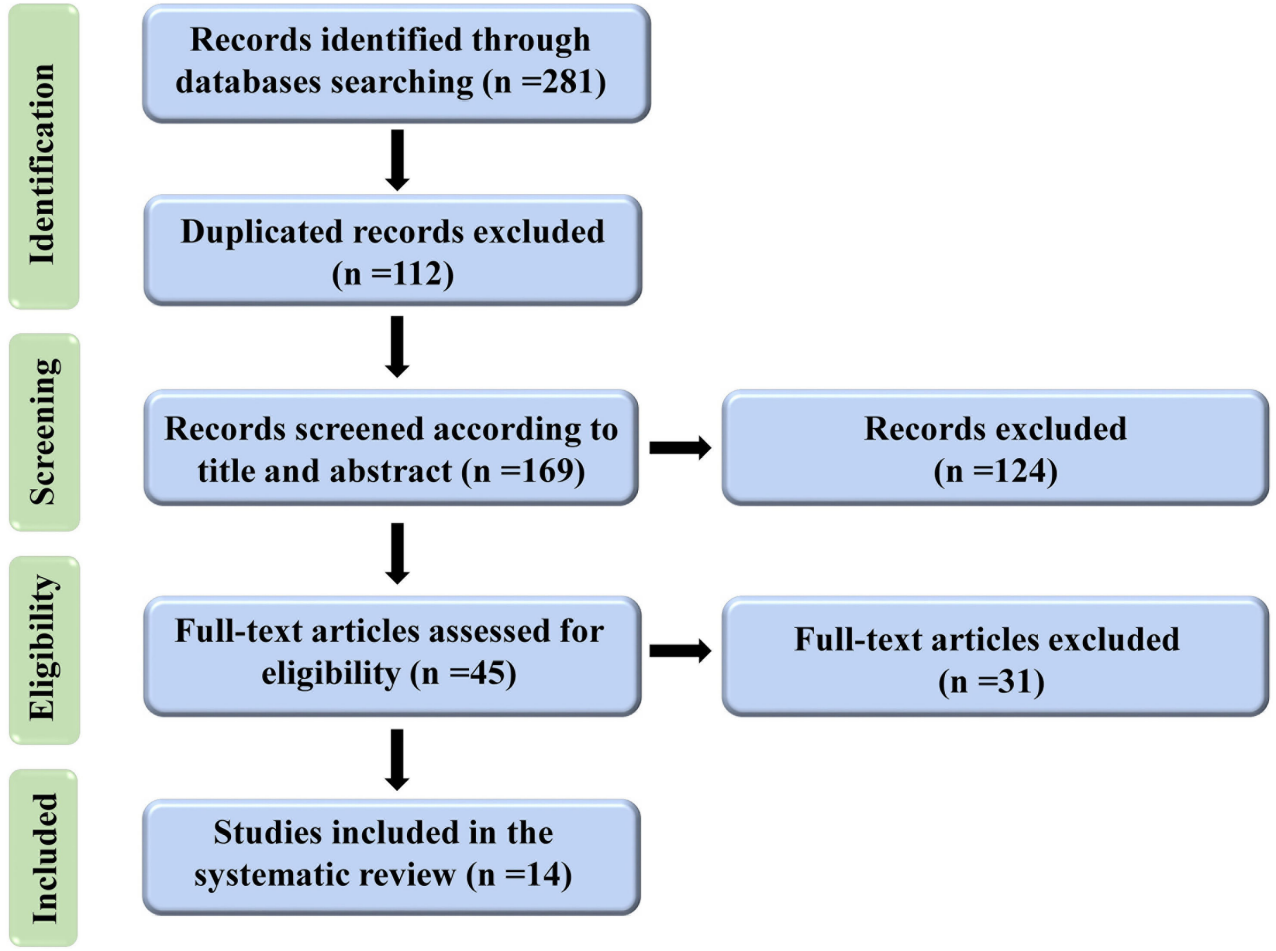
Flow diagram representing the selection process for this study.

Until June 2022, 281 articles were found after a thorough search of electronic databases. After removing duplicated articles (n=112), 169 articles were screened in titles and abstracts, and 124 were eliminated. For further evaluation of their full text, 45 articles met the (inclusion and exclusion) criteria. In the end, 14 papers were eligible for inclusion in the systematic review. **Figure 1** depicts the process of conducting a literature search and screening.

### 3.2 Data extraction

The data of each article includes 1) The first author and year of publication, 2) Models (*in-vitro* or *in-vivo*) and duration, 3) Diabetes induced agent (dosage) and route of administration, 4) Outcomes of reproductive system impairment, 5) Melatonin dosage & route of administration/duration of administration, 6) Melatonin administration outcomes, were independently extracted and organized by MA and BB, entered in **Table 1** and checked by HHA.

**Table 1:** The summary of *in-vivo* and *in-vitro* studies in systematic literature review.

Author & year	Models (Tissue) & Duration	Diabetes induced agent (dosage) & route of administration	Outcomes of reproductive system impairment	Melatonin dosage & route of administration/duration of administration	Melatonin administration outcomes
Du et al., 2018 (3)	*In-vivo* – Mice (Testes & epididymis) & 2 weeks	STZ)100 mg/kg) & ip	↓Body weight, ↓Leydig cells & SSCs, ↑Thickness of the basement membrane in seminiferous tubules, ↓Integrity of epididymal duct wall, ↓sperm density, ↓PCNA protein level, ↑Apoptosis rate, Analysis of the gene expressions: ↑P53, ↓Bcl2, ↑Caspae3, ↑Caspase 12, ↑Grp78, ↑CHOP, Analysis of the protein expression: ↓Bcl2/Bax, ↑Grp78, ↑CHOP, ↑Testicle’s CSF1 mRNA expression & protein level s	10 mg/kg/po & 2 weeks	↑Leydig cells & SSCs, ↓Thickness of the basement membrane in seminiferous tubules, ↑Integrity of epididymal duct wall, ↑sperm density, ↑PCNA positive cells, Analysis of the gene expressions: ↓P53, ↑Bcl2, ↓Caspase 3, ↓Caspase 12, ↓Grp78, ↓CHOP, Analysis of the protein expression: ↓Grp78, ↑Testicle’s CSF1 mRNA expression & protein levels
	*In-vivo* – Mice (Testes & epididymis) & 8 weeks		↓Body weight, ↑congestion, ↓Leydig cells & SSCs, ↑Thickness of the basement membrane in seminiferous tubules, ↓sperm density, ↑Abnormal sperms, ↓PCNA protein level, ↑Apoptosis rate, Analysis of the gene expression: ↑P53, ↓Bcl2, ↑Caspase 3, ↑Caspase 12, ↑Grp78, ↑CHOP, Analysis of the protein expression: ↑P53, ↓Bcl2/Bax, ↑Caspase 3, ↑Grp78, ↑CHOP, ↑Testicle’s CSF1 mRNA expression & protein level	10 mg/kg/po & 2 weeks	↑Body weight, ↓Mild puffiness, and congestion, ↑Leydig cells & SSCs, ↓Thickness of the basement membrane in seminiferous tubules, ↑Integrity of epididymal duct wall, ↑sperm density, ↓Abnormal sperms, ↑PCNA positive cells, Analysis of the gene expression: ↓P53, ↑Bcl2, ↓Caspase 3, ↓Caspase 12, ↓Grp78, Analysis of the protein expression: ↓P53, ↑Bcl2/Bax, ↓Caspase 3, ↓Grp78, ↓CHOP, ↑Testicle’s CSF1 mRNA expression & protein level
	*In-vitro* – MLTC-1	20 mM glucose	↓Cell viability, ↓BrdU positive cells, ↑TUNEL positive cells, ↑ Gene expression level of p53, p21, GRP78, CHOP, caspase 3 & Caspase12, ↓ Gene expression level of Bcl2, ↑Protein expression of P53, Caspase3 & CHOP, ↓Protein expression of Bcl2/Bax,↑G2/M phase arrest of MLTC-1	1 μM	↑Cell viability, ↓Apoptotic rate, ↑BrdU positive cells, ↓TUNEL positive cells, ↓Gene expression level of p53, p21, GRP78, CHOP, & Caspase12, ↑Expression level of Bcl2, ↓Protein expression of P53, Caspase3 & CHOP, ↑Protein expression of Bcl2/Bax, ↓G2/M phase arrest of MLTC-1
Costa et al., 2015 (31)	*In-vivo* – Rat (Testes & epididymis) & 1 week	STZ)4.5 mg/100 g) & ip	↓Body weight, ↓Frequency of seminiferous tubule lumen & ↑Seminiferous epithelium, ↑macrophage population, ↓Sperm counts and transit time induced in caudal segment of epididymis, ↓Number of motile spermatozoa with progressive movement & ↑Immotile sperm, ↓Serum testosterone level, ↑AR expression	10 µg/kg/po & 10 weeks started 9 weeks before diabetes induction	Preserved relative frequency of the main tissue compartments of testes, ↓macrophage population, ↑Sperm counts and transit time induced in caudal segment of epididymis, ↑Sperm motility, ↑Serum testosterone level
	*In-vivo* – Rat (Testes & epididymis) & 8 weeks		↓Body weight, ↓Epididymal wet weight, ↑Leydig cells volume with nuclear atrophy, ↑Histological abnormalities, ↓Sperm counts and transit time induced in the caudal segment of the epididymis, ↓Number of motile spermatozoa with progressive movement & ↑immotile sperm, ↓Serum testosterone level	10 µg/kg/po & 17 weeks started 9 weeks before diabetes induction	↑Epididymal wet weight, ↓Leydig cells volume, ↓Histological abnormalities, ↑Sperm motility, ↑Serum testosterone level
Oliveira et al., 2015 (32)	*In-vivo* – Rat (Testes & epididymis) & & 2 weeks	STZ)120 mg/kg) & ip	↓Body weight, ↓Testis & epididymis masses, ↓Plasma testosterone levels, ↓POMC mRNA levels, ↓Hypothalamic kisspeptin-1 mRNA levels	0.2 mg/kg/po & 2 weeks	No improvement
Akman et al., 2015 (33)	*In-vivo* – Rat (Testes) & 15 days	In first day alloxann (120 mg/kg) + In second day alloxan (120 mg/kg) & ip	↓Body weight, ↑MDA level, ↓GSH level & SOD activity, ↓Johnsen criteria, ↓Number of Progressively Motile Sperm, ↑Premature AR rate, ↑8OHdG immunopositive cells	10 mg/kg/ip & 15 days	↑Body weight, ↓MDA level, ↑GSH level & SOD activity, ↑Johnsen criteria, ↓8OHdG immunopositive cells
	*In-vivo* – Rat/(Testes) & 45 days		↓Body weight, ↑MDA level, ↓GHS level & SOD activity, ↓Johnsen criteria, ↓Sperm concentration, ↓Number of Progressively Motile Sperm, ↑Premature AR rate, ↑8OHdG immunopositive cells	10 mg/kg/ip & 45 days	↑Body weight, ↓MDA level, ↑GSH level & SOD activity, ↑Johnsen criteria, ↓8OHdG immunopositive cells
Guneli et al., 2008 (34)	*In-vivo* – Rat (Testes) & 5 days.	STZ (45 mg/kg) & ip	↓Body weight, ↑Disrupted seminiferous tubule structure, ↓Spermatogenic cells, ↑Thickness of STBM, ↓Seminiferous tubule diameter & Johnsen’s criteria values, ↑TUNEL positive Cells, ↑PMNs	10 mg/kg/ip & 5 days	↑Normal seminiferous tubules, ↑Spermatogenic cells, ↓Thickness of STBM, ↑Seminiferous tubule diameter & Johnsen’s criteria values, ↓TUNEL positive cells, ↓PMNs
Armagan et al., 2006 (35)	*In-vivo* – Rat (Testis) & 8 weeks	STZ (35 mg/kg) & ip	↓Body weight, ↓CAT activity, ↑MDA level & SOD activity	10 mg/kg/ip & 8 weeks	↑CAT activity, ↓ SOD activity
Alves et al., 2020 (36)	*In-vivo*–Rat (Testis) for 20 days	STZ (60 mg/kg) & ip	↓Body weight, ↓testes weight, ↓Mean height of seminiferous epithelium, ↓Tubular diameter, ↑TBARS level, ↓GSH content, ↑IL-6 & TNF-α levels, ↓Serum testosterone level, ↓AR	10 mg/kg & po for 20 days	↑Body weight, ↑testes weight, ↑Mean height of seminiferous epithelium, ↑Tubular diameter, ↓TBARS level, ↑GSH content, ↓TNF-α & IL-6 levels, ↑Serum testosterone level, ↑AR
Gobbo et al., 2015 (37)	*In-vivo* – Rat (Prostate) & 1 week	STZ (4.5 mg/100g) & ip	↓Body weight, ↓Prostate weight, ↓Cell proliferation, ↑Apoptosis, ↓Serum testosterone level, ↓AR positive cells, ↑MTR1B	10 μg/kg/po & 9 weeks started 8 weeks before induction of diabetes	↑Prostate weight, ↓MTR1B
	*In-vivo* – Rat (Prostate) & 8 weeks		↓Body weight, ↓Prostate weight, ↓Cell proliferation, ↑Apoptosis, ↓Expression of PCNA, ↓Serum testosterone level, ↑AR positive cells, ↑MTR1B	10 μg/kg/po & 16 weeks started 8 weeks before induction of diabetes	↑Cell proliferation, ↓Apoptosis ↑Expression of PCNA, ↑Serum testosterone level, ↓AR positive cells, ↓MTR1B
Gobbo et al., 2015 (37)	*In-vivo* – Rat (Prostate, testis, and epididymis) & 1 week	STZ (4.5 mg/100g) & ip	↓Body weight, ↓Prostate weight, ↑Prostate GST & GPx activity, ↓Epididymis weight, ↑Epididymis GPx activity, ↑Testis GST	10 μg/kg/po & 9 weeks started 8 weeks before induction of diabetes	↑Prostate weight, ↓GPx activity
	*In-vivo* – Rat (Prostate, testis, and epididymis) & 2 months		↓Body weight, ↓Prostate weight, ↑Prostate CAT & GST activity, ↑Prostate LPO, ↓Epididymis weight	10 μg/kg/po & 16 weeks started 8 weeks before induction of diabetes	↓Prostate CAT activity & GST activity, ↓Prostate LPO, ↑Epididymis weight
Gobbo et al., 2017 (38)	*In-vivo* – Rat/(Prostate) & 1 week	STZ (40 mg/kg) & ip	↓Body weight, ↓Prostate weight, ↓Absolute frequency of epithelium, Thinner and interrupted smc layers, ↑5MeC-positive cells, ↓Androgen levels,	10 μg/kg/po & 9 weeks started 8 weeks before induction of diabetes	↑Prostate weight, ↑Acinar epithelium, thickness of smc layer, & collagen fibers, ↓5MeC-positive cells
	*In-vivo* – Rat/(Prostate) & 2 months		↓Body weight, ↓Prostate weight, Atrophied acinar epithelium and smc layer, ↓collagen fiber distribution, ↑Density of acinar atrophy, ↑Epithelial metaplasia, ↑PIA & PIN, ↑Prostatitis, ↑5MeC-positive cells, ↓Androgen levels	10 μg/kg/po & 16 weeks started 8 weeks before induction of diabetes	↓smc atrophy, ↑collagen fibers distribution, ↓Density of acinar atrophy, ↓Epithelial metaplasia, ↓PIA & PIN, ↓Prostatitis, ↑Androgen levels
Qiu et al., 2012 (39)	*In-vivo* – Rat/(Cavernosum) & 8 weeks	STZ (60 mg kg) & ip	↓Endothelial density & smooth muscle/collagen ratio	10 mg/kg·/ip & 8 weeks	↑Endothelial density
Paskaloglu et al., 2004 (40)	*In-vivo* – Rat/(Corpus cavernosum) & for 8 week	STZ (60 mg kg) & ip	↑MDA & ↓GSH level	10 mg/kg & ip for 8 weeks	↓MDA & ↑GSH level
Sahan et al, 2020 (41)	*In-vivo* – Rat/(Cavernosal tissue) & 10 weeks	STZ (60 mg kg) & ip	↑8OHdG & MDA levels, ↓NOS activity, ↓cGMP, ↑caspase-3 activity, ↓SIRT-1 & e-NOS protein expression, ↑Vascular congestion, sinusoidal damage & cytoplasmic vacuolisation in the endothelial cells, ↓Serum testosterone level	10 mg/kg & ip for 10 weeks	↓8OHdG & MDA levels, ↑NOS activity, ↑cGMP, ↓caspase-3 activity, ↑SIRT-1 & e-NOS protein expression, ↓vascular congestion & preserved the sinusoidal structure, ↑Serum testosterone level
Nayki et al., 2016 (42)	*In-vivo* – Rat/(Ovary) & 8 weeks	STZ (50 mg/kg/day for 5 days) & ip	↑MDA levels, ↓SOD activity, ↑TOS, ↓TAS, ↑Expressions of NF–kB & caspase-3, ↑Stromal fibrosis, ↑Follicular degeneration & hemorrhage	20 mg/kg·/ip & 3 weeks	↓MDA levels, ↑SOD activity, ↓TOS, ↑TAS, ↓Expression of NF–kB & caspase-3, ↓Stromal fibrosis, ↓Follicular degeneration & hemorrhage

↑, Increase; ↓, Decrease; &, And; ip, Intraperitoneal; po, Per os; MDA, malondialdehyde; SOD, superoxide dismutase; GSH, glutathione; GPx, glutathione peroxidase; GST, glutathione-S-transferase; SOD, superoxide dismutase; 8OHdG, 8-hydroxydeoxyguanosine; LPO, lipid peroxidation; TBARS, Thiobarbituric acid reactive substances; CHOP, C/EBP homologous protein; Bcl 2, B-cell lymphoma 2; Bax, BCL2-associated X protein; TNF-α, tumor necrosis factor-alpha; IL-6, interleukin 6; NF-κB, Nuclear Factor Kappa B; NOS, nitric oxide synthesize; e-NOS, endothelial nitric oxide synthesize; MTR1B, Melatonin Receptor 1B; STBM, seminiferous tubule basement membrane; PIA, prostate intraepithelial neoplasia; PIN Proliferative inflammatory atrophy; CSF1, Colony Stimulating Factor 1; AR, androgenic receptors; PCNA, proliferating cell nuclear antigen; TAS, total antioxidant status; TOS, total oxidant status

### 3.3 The studies are categorized into male and female areas:

#### 3.3.1 Studies on diabetic-induced male reproductive damage

##### 3.3.1.1 Epididymis

The results of the studies indicated that experimental DM significantly reduced the epididymis weights or masses (31, 32, 37). In comparison, melatonin administration could increase the weight of epididymis (31, 32). Induction of DM caused a mild increase in glutathione peroxidase (GPx) activity than the control group that was not affected by melatonin treatment (37). Also, DM induced degeneration of germ cells in the ductal lumen, induced atrophy of epididymal cauda, and disturbed the epididymal duct wall (3, 31). The diabetic subjects treated with melatonin showed minor histological changes (3).

##### 3.3.1.2 Testis

According to the results, although DM did not meaningfully affect testes’ weights in most reports (3, 31, 35, 37), it significantly reduced testis weights in two studies (3, 32). Treatment with melatonin increased testes weight in a study (36). Evaluation of the results showed that DM significantly increased the levels of thiobarbituric acid reactive substances (TBARs), malondialdehyde (MDA), 8-hydroxydeoxyguanosine (8-OHdG), as well as (glutathione-S-transferase) GST activity, reduced glutathione (GSH) levels, and catalase (CAT) activity, and caused an imbalance (elevation or reduction) in superoxide dismutase (SOD) activity (33, 35–37). In comparison, melatonin treatment could balance the parameters mentioned above in diabetic subjects (33, 35, 36). DM-induced hyperglycemia significantly up-regulated 78-KD glucose-regulated protein (Grp78), C/EBP homologous protein (CHOP), caspase-12, P53, and caspase-3 genes, increased protein expressions of Grp78, CHOP, apoptotic proteins such as P53 and caspase-3, elevated tumor necrosis factor-alpha (TNF-α), and interleukin-6 (IL-6) also reduced ratio of Bcl-2/Bax. Treatment with melatonin could mainly normalize these altered parameters (3, 36). Histological examination in the studies showed various abnormalities in testis tissue, such as congestion, loss of Leydig cells and spermatogonial stem cells (SSCs), increased seminiferous epithelium and Leydig cells volume with nuclear atrophy, reduction in the tubular diameter and the height of the seminiferous epithelium, the elevation of the Seminiferous Tubules Basement Membrane (STBM) thickness, and disorganization and depletion of germ cells and vacuolization of Sertoli cells in the diabetic group (3, 31, 33, 34, 36). The diabetic subjects treated with melatonin showed less histological injury in testis tissue (3, 31, 33, 34, 36).

##### 3.3.1.3 Sperm alterations

Evaluation of the results showed a reduction in sperm concentration or density, the number of progressively motile spermatozoa, and transit time in the caudal segment of the epididymis, as well as an increase in sperm abnormalities, premature acrosome reaction rates, and immotile sperms in un-treated diabetic groups (3, 31, 33). However, treatment with melatonin was associated with increased sperm count and transit time and improvement of sperm abnormalities and motility (3, 31).

##### 3.3.1.4 Prostate

The results indicated decreased prostate weight in diabetic groups compared to the control group. Melatonin treatment reversed prostate weight to normal (37, 38). Also, experimental DM caused elevation in GPx, CAT, GST, and MDA content balanced by melatonin treatment (37). Moreover, DM caused various histological abnormalities indicated by the reduced absolute frequency of epithelium, thinner and interrupted smooth muscle cell (SMC) layers, increased apoptotic cells, neoplastic lesions, and metaplasia. Melatonin administration mainly improved the alterations above (37, 38).

##### 3.3.1.5 Corpus Cavernosum

Hyperglycemia causes alterations in various parameters in the cavernosal tissue. It significantly increased MDA, 8-OHdG levels, and caspase-3 activity while reducing GSH content, cyclic guanosine monophosphate (cGMP) level, sirtuin-1 (SIRT-1), and endothelial nitric oxide synthase (e-NOS) protein expression in diabetic rats. In contrast, melatonin administration markedly balanced the changes in these parameters (40, 41). The assessment of pathological changes in the tissue represented a reduction of endothelial density and smooth muscle ratio to collagen, cytoplasmic vacuolization in the endothelial cells, and vascular congestion mitigated by co-treatment with melatonin (39, 41).

#### 3.3.2 Studies on diabetic-induced female reproductive damage

Based on our search strategy, just one study was found in this area.

##### 3.3.2.1 Ovary

The present investigation demonstrated that untreated DM caused increased MDA level, decreased SOD and CAT activity, imbalanced total oxidant and antioxidant status, and elevated Nuclear Factor Kappa B (NF-κB) and caspase-3 levels. The results indicated that melatonin could reverse the alterations in the parameters mentioned above. Moreover, histological findings represented stromal fibrosis, increased follicular degeneration, and hemorrhage normalized by melatonin co-treatment (42).

#### 3.3.3 Effects of melatonin on sexual hormones and receptors

Hyperglycemic rats were reported to have altered steroidogenesis, exhibiting decreased serum testosterone levels (31, 36, 37, 41). Melatonin treatment prevented the drop in circulating levels of testosterone due to DM (31, 36, 37, 41).

Diabetes induction resulted in decreased androgen receptors (AR) in the testis. In contrast, melatonin could moderately increase the value (36). Based on another study, the rate of ARs elevated in the untreated DM group was non-significantly reduced after melatonin treatment, while after eight weeks, melatonin increased ARs in testis (31). In a study, the AR rate in the prostate decreased in the short period of DM (1 week), whereas it significantly increased in long-term DM (8 weeks). Melatonin abolished the increase of ARs in long-term DM (37).

## 4 Discussion

**Figure 2:**
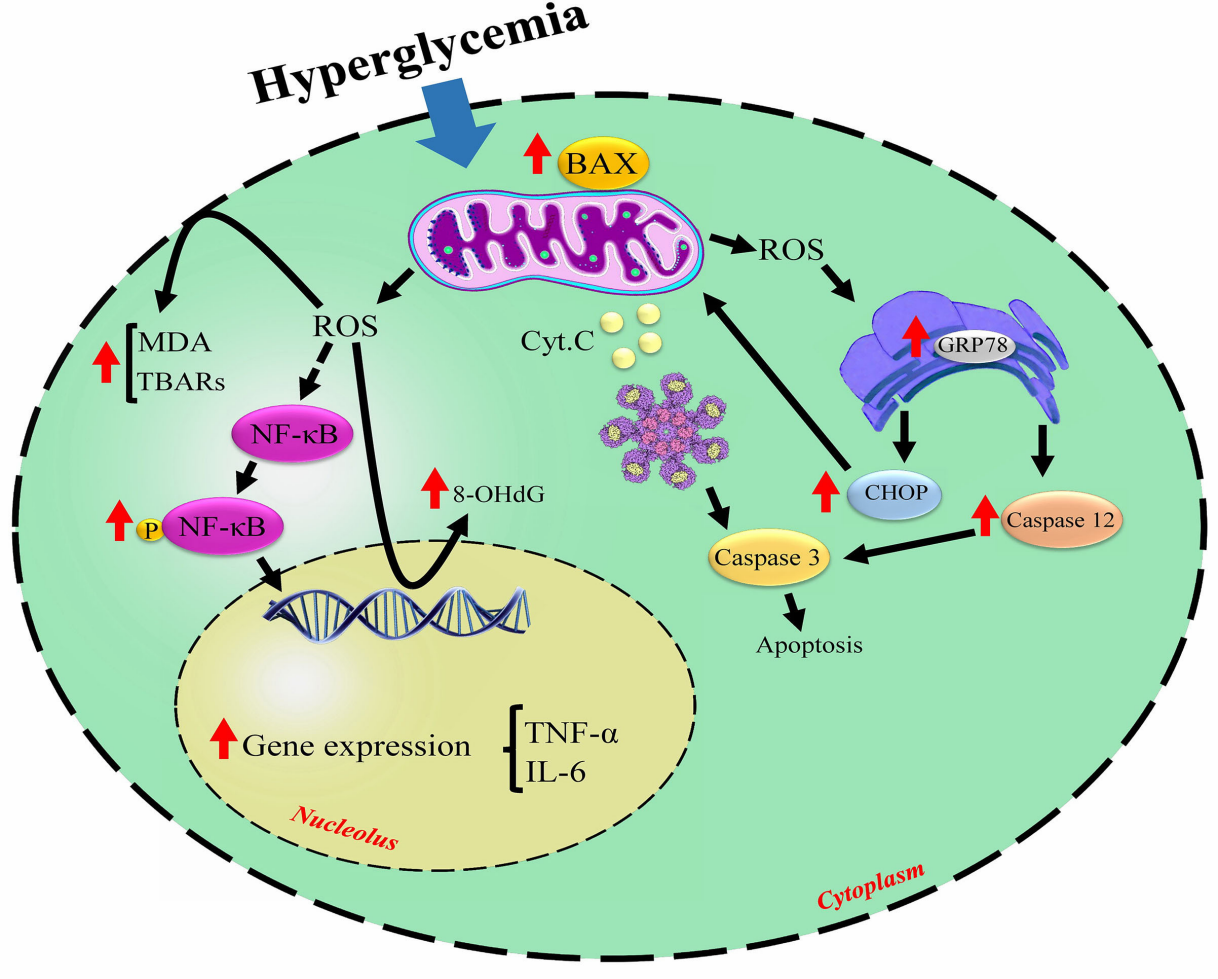
The noticed mechanisms involved in diabetes-related cellular damage. Hyperglycemia triggers excessive ROS production leading to the cell membrane and DNA damage, induces apoptosis signaling pathways, and causes inflammation. ↑increased by hyperglycemia; ROS, reactive oxygen species; MDA, malondialdehyde; TBARs, thiobarbituric acid reactive substances; NF-κB, nuclear factor-kappa-β; IL-6, interleukin 6; TNF-α, tumor necrosis factor-alpha; GRP78, 78-KD glucose-regulated protein; CHOP, C/EBP homologous protein; Cyt c, cytochrome C.

The present review evaluates the mechanisms involved in DM-induced damage to reproductive organs and the effect of melatonin administration on the reproductive system affected by hyperglycemia. The included studies used STZ or alloxan, leading to type 1 diabetes. Some of the crucial mechanisms involved in DM-induced reproductive injury are illustrated in the **Figure 2**.

Based on the evidence, DM can adversely affect various organs, among which reproductive injury has currently attracted much attention (43). Based on several findings, melatonin has roles in testicular function, the self-renewal of mouse SSCs, spermatogenesis, and oogenesis, demonstrating its beneficial effect on reproduction activity. Furthermore, different models evaluated melatonin as a potent antioxidant and anti-inflammatory molecule in mitigating reproductive damage (44, 45). In the following parts, we will focus on the mechanisms of melatonin on the reproductive system malfunction during the diabetic condition.

### 4.1 Melatonin as an antioxidant agent

In a physiological state, free radicals, as products of cell physiological processes, are maintained at equilibrium by antioxidant system activity (46). While in pathological conditions, accumulating large amounts of free radicals can exceed the antioxidant defense capacity, leading to oxidative stress (47, 48). A large body of evidence has demonstrated that an increased amount of ROS and weakened antioxidant defense caused by hyperglycemia result in oxidative stress causing damage to various cell macromolecules such as proteins, lipids, and nucleic acids (8, 49). Hyperglycemia causes severe oxidative stress through glucose auto-oxidation, multiple proteins glycation, and polyol pathway induction (46). In this condition, mitochondria are the primary oxidative stress sources. Indeed, during mitochondrial oxidative metabolism, the excess oxygen is converted to oxygen free radical (O^-^), known as the main ROS that is transformed to other reactive species such as superoxide anion $\left(O_{2}^{-}\right)$, hydroxyl (OH^-^), hydrogen peroxide (H_2_O_2_), and peroxynitrite (ONOO^-^) contributing to oxidative or nitrosative stress progress (50, 51). DM-related oxidative stress can induce malfunction in genital organs and subsequent infertility (31). The detrimental effect of excessive ROS on various reproductive tissues, including testis, prostate, and corpus cavernosum, is associated with lipid peroxidation characterized by elevated TBARS and MDA levels leading to cell membrane disruption (36, 37, 41).

Moreover, severe oxidative stress potentially contributes to DNA damage indicated by increased cellular 8-oxo, 2’-deoxyguanosine (8-oxodG), as a sensitive biomarker (52). Oxidative stress in sperm may interfere with membrane function, damage DNA, and negatively affect motility (33). GSH, a tripeptide molecule, potentially protects the cell against reactive species. Oxidative stress caused by hyperglycemia can deplete testis tissue GSH levels due to its oxidation to GSH disulfide (GSSG) (33, 36). Also, the alterations in the intracellular antioxidant enzymes (CAT, SOD, GPx, GST) involved in tissue protection may occur in response to oxidative stress resulting in tissue damage (33, 35, 37). Findings of a study have demonstrated that oxidative stress induced by experimental DM can make an antioxidant system response in male reproductive organs, particularly in prostate tissue that shows more vulnerability to hyperglycemia than the testis and epididymis, possibly due to different histopathologic characteristics and patterns of antioxidant enzymes expression (37). However, these findings contrasted with other research that found substantial alterations in testicular antioxidant enzymes in diabetic rats (33, 35). While the exact reason for these discrepancies is unclear, it may partially be explained by differences in the type and dosage of the chemicals used for induction of diabetes, the experiments’ methodology, and length.

Melatonin is a neurohormone with potent antioxidant action that readily crosses the cell membranes and biological barriers such as the brain and testis blood barriers (27, 34). By route, melatonin administration causes a fast rise in blood melatonin concentrations. It can reach a high level in mitochondria, the leading source of free radicals. Melatonin’s extensive subcellular distribution may minimize oxidative damage in the cell’s lipid and aqueous environments. Melatonin has an advantage over other antioxidants since it more slowly infiltrates cells (21). The antioxidant effect of melatonin is due to its direct and indirect antioxidant action. This molecule and its metabolites scavenge and detoxify various free radicals like superoxide anion, singlet oxygen, peroxyl radicals, and peroxynitrite anion. It also mitigates oxidative stress by enhancing antioxidative defense capacity, and regulating the expression and activity of multiple antioxidant enzymes and GSH content (53). Melatonin through melatonin receptor 1 (MT1) and interaction with calmodulin can inactivate the nuclear ROR-alpha melatonin receptor, a nuclear receptor, resulting in a modulation of antioxidant enzyme gene expression (54). So, melatonin can normalize the altered activity of SOD as a crucial enzyme in converting the superoxide anion into H_2_O_2_ and molecular oxygen.

Moreover, it can balance the hyperglycemia-induced changes in CAT and GPx as the enzymes neutralize H_2_O_2_, without which the high level of H_2_O_2_ will damage lipid membranes, causing the production of excessive MDA. This antioxidant molecule can normalize GST activity as an enzyme catalyzing the conjugation of GSH to various electrophilic molecules and possibly a diabetes-induced specific marker of prostate oxidative stress (37). GSH is regenerated by reducing GSSG due to the glutathione reductase (GR) enzyme’s function *via* nicotinamide adenine dinucleotide phosphate (NADPH) as a co-factor. Melatonin, possibly through its antioxidant action, up-regulation of cell GSH, and increased g-glutamylcysteine, can improve cellular GSH content (33, 36, 40). The antioxidant ability of melatonin was demonstrated when used to inhibit lipid peroxidation in testis tissue of diabetic rats. This decrease in lipid peroxidation is most likely due to the direct neutralization of free radicals and the indirect antioxidant effect through modulating the GSH levels and activity of CAT and SOD, resulting in less testis histopathological damage (33, 35, 36). Melatonin can restore sperm parameters and motility (3, 31). Although the indicators of oxidative stress in the diabetic rat epididymis did not alter (37), it is plausible that melatonin has a direct antioxidant effect on spermatozoa (55).

Also, melatonin restores the activity of antioxidant enzymes (CAT, GPx, GST) and reduces LPO in the prostate of diabetic rats, demonstrating its potent antioxidant action in this organ (37). Noteworthy, melatonin protects cavernosal tissue in type 1 diabetic rats by increasing GSH levels, reducing oxidative tissue damage and the harmful impact of free radicals on nitric oxide activity, contributing to corporal function improvement (40, 41).

### 4.2 Anti-apoptotic action of melatonin

Apoptosis is a controlled cell death playing a crucial role in maintaining cellular homeostasis (56, 57). Any disturbances regulating this pathway lead to cellular abnormalities and diseases (58, 59). Apoptosis can initiate in three main ways: extrinsic, intrinsic mitochondrial, and intrinsic endoplasmic reticulum pathways. Evidence suggests that glucose autooxidation-induced ROS production may be a driving force for activating the mitochondria-dependent path (60, 61). Indeed, the imbalance between anti-apoptotic proteins such as Bcl-2, B-cell lymphoma-extra-large (Bcl-xL), and also pro-apoptotic agents, including Bcl-2 associated agonist of cell death (Bad), BH3 interacting-domain death agonist (Bid), and Bcl-2-associated X (Bax) may occur due to activation of p53 induced by the high level of ROS (62, 63). These alterations lead to disruption in the outer mitochondrial membrane and release of cytochrome c involved in the apoptosome formation, a complex consisting of apoptotic protease activating factor-1 (Apaf1) and procaspase-9, which cleave procaspase-9 into caspase-9. Caspase-9 can finally activate caspase-3, which starts DNase and degrades DNA (64, 65). Based on the evidence, melatonin, through affecting its receptors, induces cellular signal transduction, leading to Bcl-2 up-regulation and re-localization to mitochondria, where Bax acts as a pro-apoptotic protein (66, 67). Notably, it prevents hyperglycemia-induced ovarian apoptosis by inducing antioxidant enzymes and inhibiting the caspase-3 pathway involved in the pathophysiology of ovarian injury (42). In cavernosal tissue, reduced caspase-3 activity reveals that melatonin might delay apoptosis by minimizing oxidative stress and improving tissue integrity (41).

The endoplasmic reticulum (ER), an organelle responsible for protein organization, sensitively reacts to homeostasis disturbance (68). Various stimuli, including nutrient restriction, altered glycosylation of proteins, and oxidative stress, are linked to the interference of proper protein folding, resulting in the accumulation of misfolded proteins and ER stress (ERS). ERS stimulates a group of signaling pathways recognized as the unfolded protein response (UPR). There are three main sensor proteins involved in UPR, including inositol-requiring protein-1 (IRE1), induced activation of transcription factor 6 (ATF6), and protein kinase RNA (PKR)-like ER kinase (PERK) that respond to the accumulation of misfolded proteins in the ER lumen and protect ER function by a reduction in protein translation rate (69). GRP78, an ER chaperone, is the primary regulator of UPR that prevents the activation of sensor proteins in resting cells (70, 71). Nevertheless, GRP78 is liberated from these transmembrane proteins in stressed cells, allowing them to activate their UPR pathways (70). A high glucose environment can shift the UPR pathway to an apoptotic program and promote the expression of the nuclear transcription factor CHOP and caspase-12 in Leydig cells (3). CHOP suppresses the production of Bcl-2, making cells more susceptible to apoptosis (72). Moreover, activated caspase-12 translocates to the cytoplasm and activates caspase-9 and, subsequently, caspase-3 playing a leading role in cellular death (73).

In diabetic conditions, melatonin can reduce the apoptosis rate induced by ERS by inhibiting Grp78, CHOP, caspase-12, and their downstream cascades. This effect of melatonin provides a more intact niche for spermatogonial stem cells to self-renew, maintaining sperm healthier for fertility (3).

### 4.3 Anti-inflammatory effect of melatonin

Inflammation is an immune system reaction to harmful factors that account for the induction of several cellular signals. Excessive generation of various pro-inflammatory cytokines during the process worsens this response (74, 75). Studies have demonstrated that DM can impair reproductive organs by inducing inflammation. In this regard, experimental DM-induced oxidative stress triggers up-regulation of nuclear factor kappa B (NF-κB), a primary regulator of inflammation (76). Indeed, oxidative stress activates IκB kinase (IKK) that is responsible for phosphorylation of inhibitor of nuclear factor kappa B (IκB-α) and activation of NF-κB, resulting in up-regulation of pro-inflammatory cytokines such as TNF-α, IL-6, interleukin 1 beta (IL1-β), and cyclooxygenase-2 (COX-2) (54, 77). According to several studies, melatonin can decrease chronic and acute inflammation. It has been shown that melatonin reduces NF-κB translocation and downregulates inflammatory cytokines by blocking the activation of IKK and IκB-α, leading to an anti-inflammatory and anti-apoptotic action (77, 78). Melatonin benefits spermatogenesis in diabetic rats by reducing inflammatory cytokines such as TNF-α and IL6 (36). This molecule mitigates the detrimental impact of diabetes-induced oxidative stress on the ovaries *via* the NF-κB pathway, leading to reduced tissue injury such as fibrosis of the stroma and follicular degeneration (42).

### 4.4 Melatonin effect on androgen synthesis

In a well-functioning hypothalamic pituitary gonadal (HPG) axis, pulses of gonadotropin-releasing hormone (GnRH) produced by the hypothalamus induce secretion of follicle-stimulating hormone (FSH) and luteinizing hormone (LH) from the pituitary gland (16). FSH and LH stimulate spermatogenesis by acting on the Sertoli and Leydig cells. FSH binds to its receptor, expressed only by Sertoli cells, increasing cyclic adenosine monophosphate (cAMP) levels and stimulating the synthesis of androgen-binding protein (ABP) and release of LH, which controls testosterone production in Leydig cells (79, 80). Spermatogenesis fails to go through the meiosis stage when testosterone or androgen receptors are absent (81). The detrimental effect of DM on testosterone production and spermatogenesis may be related to the disturbed production and release of gonadotropins due to the link between the neurological and endocrine systems, causing structural alterations in Sertoli and Leydig cells (16, 82). Besides, the marked testosterone decrease in experimental diabetes can be linked to the lack of insulin because insulin can affect Leydig cells as a paracrine factor (36). Notably, melatonin modulates androgen release in Leydig cells *via* binding to a melatonin membrane receptor. In this regard, steroid production is accelerated when phosphorylated cyclic AMP response element-binding protein (CREB) binds to the steroidogenic acute regulatory protein (StAR) promoter’s cAMP response element (83). Some investigations have shown that rising androgen levels are predominantly due to melatonin’s stimulatory impact on the steroidogenic enzyme 3-hydroxysteroid dehydrogenase, an enzyme in the adrenal gland that catalyzes the synthesis of androstenedione from dehydroepiandrosterone (DHEA) (84). Melatonin can also affect the activity of the HPG axis by influencing GnRH production *via* binding sites in the suprachiasmatic nucleus, hypothalamic premammillary, and mediobasal hypothalamus (85). Besides, it has been manifested that melatonin improves spermatogenesis by improving glucose metabolism, facilitating glucose transport activity, and stimulating the Krebs cycle in Sertoli cells (86). The metabolism of Leydig cells may be similarly enhanced by exogenous melatonin in the Type 1 DM model, leading to the improvement of steroidogenesis. However, melatonin’s effect on Leydig cells must be verified (31). Melatonin ingestion increases AR expression in the testes, suggesting altering androgen signaling. The improvement in testosterone production might be explained by the enhanced AR expression generated by melatonin therapy since experiments with null animals for this receptor revealed decreased levels of 17β- hydroxysteroid dehydrogenase and 3β-hydroxysteroid dehydrogenase expression (31, 87).

### 4.5 Future research prospects

Persistent hyperglycemia due to diabetes is accompanied by long-term injury, malfunction, and failure of various organs (88). Diabetes is also associated with reproductive impairment in both genders. Its negative effect on reproduction can be substantial, as seen by decreased fertility and reproductive failure (89). The studies show that precise glycemic control combined with antioxidant treatment can help reduce the risk of harmful diabetic outcomes (2). Studies have implied melatonin’s role in modulating several signaling pathways and indicated its preventive and therapeutic effects in various diseases, such as DM. Many animal and human research studies have shown the anti-diabetic effect of melatonin and its leading role in reducing DM-related complications, including cardiomyopathy, nephropathy, retinopathy, and neuropathy (27, 90). The present study demonstrated that melatonin administration could be associated with declining oxidative stress, blocking the apoptosis signaling pathway, modulating endoplasmic reticulum function, and reducing inflammation in various reproductive components (3, 32, 33, 35–38). Also, according to most of the findings, melatonin can positively affect androgen levels in diabetic male subjects (31, 36, 37, 41).

Nevertheless, It should be noticed that in experimental studies, the factors including disease length, glycemic levels, and the direct destructive effect of the chemicals used to induce diabetes on various tissues may conceal the true impact of diabetes on fertility during the investigation. The use of the induced type 1 diabetic animal models in the present studies may not accurately mimic the pathologic processes of the other form of diabetes. Indeed, type I and type II diabetic studies differ in animal models used in various aspects, including insulin resistance and body mass index. Besides, Type I diabetes is less common than type II diabetes in the community. Additionally, the included studies do not explain the interaction between DM and melatonin on females’ sexual hormones, and this aspect of the story remains unexplored. Therefore, further research on mechanisms exerted by melatonin in diabetic reproductive dysfunction in multiple elements of pre- and clinical studies will improve our insights.

## 5 Conclusion

Overall, DM is responsible for abnormalities in male and female reproductive system components, including ovary, corpus cavernosum, testis, epididymis, prostate, sperm concentration and motility, and testosterone level through several mechanisms. During the hyperglycemic condition, oxidative stress, apoptosis, inflammation, histological injury, and disturbance in androgen hormone will affect normal cells of the reproductive system leading to fertility disorders. Melatonin supplementation positively affects the reproductive tissues through several mechanisms. Melatonin neutralizes reactive species, indirectly modulates antioxidant enzymes, balances apoptotic and anti-apoptotic agents, downregulates the factors related to endoplasmic reticulum stress, inhibits pro-inflammatory cytokines, and normalizes testosterone levels. According to the present results, melatonin protects the reproductive organs during DM-induced hyperglycemia in non-clinical models, which must be proven in a clinical setting.

## Data availability statement

The original contributions presented in the study are included in the article/**Supplementary Material**. Further inquiries can be directed to the corresponding authors.

## Author contributions

MAr gave the idea, extracted data, and drafted some parts of the manuscript. BB did the literature search and screening, extracted data, and drafted some parts of the manuscript. HH-A did the literature search and screening, prepared figures, and supervised the study. RF, MAt, SH, MS, and MHS drafted some parts of the manuscript and provided comprehensive revision and editing. MAb conceived the study, comprehensively edited the manuscript, and supervised the process. All authors read and approved the final version.

## Acknowledgments

The authors wish to thank Tehran University of Medical Sciences and Ardabil University of Medical Sciences for providing the full text of the articles needed. The authors wish to thank INSF for the general Seat Award directed to MAb.

## Conflict of interest

The authors declare that the research was conducted in the absence of any commercial or financial relationships that could be construed as a potential conflict of interest.

## Publisher’s note

All claims expressed in this article are solely those of the authors and do not necessarily represent those of their affiliated organizations, or those of the publisher, the editors and the reviewers. Any product that may be evaluated in this article, or claim that may be made by its manufacturer, is not guaranteed or endorsed by the publisher.
