# Supplementary material for: A systematic review on the role of melatonin and its mechanisms on diabetes-related reproductive impairment in non-clinical studies
Source: Front Endocrinol (Lausanne). 2022 Oct 11;13:1022989. doi: 10.3389/fendo.2022.1022989 (PMC9592976; doi:10.3389/fendo.2022.1022989)
Supplement: Supplementary file 1 [file DataSheet_1.docx]

The search terms used for the study's purpose were Melatonin AND Diabetes* OR Hyperglycemia OR NIDDM OR IDDM AND Testes OR Testicle OR Testis OR Testicular OR Genital* OR Reproductive OR Sperm* OR Follicle* OR Sertoli OR Gonadotropin-Releasing Hormone OR Gonadoliberin OR LH Releasing Hormone OR Gonadorelin OR FSH Releasing Hormone OR GnRH OR Prolactin OR PRL OR Follitropin* OR Follitropin releasing factor OR Gonadorelin derivative OR Prostate OR Male genital system OR Female genital system OR Ovary OR Uterus OR Uretri* OR Womb OR Oocyte OR Ovocyte OR Ovarian OR Ovaries OR Epididymis OR Semen OR Gametes OR Gametogenese OR Reproductive cells OR Fertilized Eggs OR Fertilized Egg OR Zygote OR Ovum OR Ova OR Endometrium OR Endometria OR fallopian tubes OR Oviduct* OR Fallopian* OR Salpinx OR Salpinges OR Estrogen* OR Progesterone OR Pregnenedione OR Testosterone OR Androtop OR Histerone OR Sterotate OR Testolin OR Testopel OR Testoderm OR Androderm OR Sustanon OR Testim OR LH OR Luteinizing hormone OR FSH OR Cervix OR Gonad OR Endometrial OR Uterine up to June 2022.
